# 4-Ferrocenylpyridine- and 4-Ferrocenyl-3-ferrocenylmethyl-3,4-dihydropyridine-3,5-dicarbonitriles: Multi-Component Synthesis, Structures and Electrochemistry

**DOI:** 10.3390/molecules170910079

**Published:** 2012-08-24

**Authors:** Elena I. Klimova, Marcos Flores-Alamo, Sandra Cortez Maya, Mark E. Martínez, Luis Ortiz-Frade, Tatiana Klimova

**Affiliations:** 1 Facultad de Química, Universidad Nacional Autónoma de México, Cd. Universitaria, Coyoacán, C. P. 04510, México D. F., Mexico; Email: mfa24s99@gmail.com (M.F.-A.); azulsacm@yahoo.com.mx (S.C.M.); mmartinez_92@yahoo.com (M.E.M.); tklimova@gmail.com (T.K.); 2 Departamento de Electroquímica, Centro de Investigación y Desarrollo Tecnológico en Electroquímica S.C. Parque Tecnológico Querétaro, Sanfandila, Pedro de Escobedo, C. P. 76703, Querétaro, Mexico; Email: laofrade@gmail.com

**Keywords:** ferrocene, 2-cyano-3-ferrocenylacrylonitrile, malononitrile, X-ray diffraction, amino(ferrocenyl)pyridine-3,5-dicarbonitriles, cyclic voltammetry

## Abstract

The reactions of 2-cyano-3-ferrocenylacrylonitrile (**1**) with malononitrile (**2**) in a MeOH/H_2_O or 2-PrOH/H_2_O medium in the presence of Na_2_CO_3_ afforded 6-alkoxy-2-amino-4-ferrocenylpyridine-3,5-dicarbonitriles **3a**,**b** (multi-component condensation) and 6-alkoxy-2-amino-4-ferrocenyl-3-ferrocenylmethyl-3,4-dihydropyridine-3,5-dicarbonitriles **4a**,**b** (multi-component cyclodimerization). Analogous reactions of **1** with **2** in an MeOH/H_2_O medium in the presence of NaOH, piperidine, or morpholine gave compounds **3a**, **4a** and 2-amino-4-ferrocenyl-6-hydroxy-, 6-piperidino- and 6-morpholinopyridine-3,5-dicarbonitriles **3c**–**e**, respectively. The structures of the compounds **3b**, **4a** and **4b** were established by the spectroscopic data and X-ray diffraction analysis. The electrochemical behaviour of compounds **3b**, **3d** and **4b** was investigated by means of cyclic voltammetry.

## 1. Introduction

Pyridine derivatives have been studied for over a century as an important class of heterocyclic compounds and they still continue to attract considerable attention due to the wide range of medicinal properties they possess, such as vasodilators, anticoagulants, hypolipidemic, tuberculostatic, antihistamine, antihypertensive, cardiovascular and gastrointestinal activities [1,2]. Pyridine systems are also found in important vitamins (PP, B_6_), alkaloids and herbicides [1].

The incorporation of one or two iron-containing ferrocene substituents into a pyridine molecule will enlarge the spectrum of valuable characteristics. In addition, ferrocene compounds are known to exhibit chemotherapeutic properties [3]. Ferrocenyl-substituted pyridines have been extensively studied as ligands, in the synthesis of non-linear optical materials, *etc*. [4,5,6]. However, their biological activities have not hitherto been studied. Various methods to prepare ferroceno-containing pyridines have been reported [7,8,9,10]. Syntheses of ferrocenylpyridines are mainly carried out via Negishi cross-coupling reactions of FcZnCl with bromopyridines [7], the condensations of 1,3-diketones with ferrocenecarboxaldehyde in the presence of AcONH_4_ [8]; the interactions of ferrocenyl-1,2-enones with 3-aminocrotononitrile [9], ethyl 3-aminocrotonate [9] or acetonitrile in the presence of Me_3_COK [10]. The interest in heterocyclic compounds bearing ferrocenyl substituents in the molecules can be traced back to the discovery of ferrocene [11,12,13,14]. This is determined by a peculiar chemical behavior of such compounds due to mutual influence of the metallocene and heterocyclic moieties. In particular, biological activities of many nitrogen heterocycles, such as quinuclidines, pyrazolines, pyrazoles, pyrimidines, tetrahydropyridazines, bearing ferrocenyl substituents, have been reported [8,15,16,17,18,19,20]. It may be expected that ferrocenylpyridines and cyano(ferrocenyl)pyridines will also prove valuable, because they possess diverse biological activity, find use as potential bio-receptor ligands [13,14,15], new drugs [16,17,18,19,20], and significant intermediates for the synthesis of important materials [21,22,23]. For these reasons, development of new compounds containing cyano and ferrocenyl groups in the pyridines is strongly desired.

Herein we report results from our investigations into reactions of the condensation of 2-cyano-3-ferrocenylacrylonitrile (**1**) with malononitrile (**2**) and of the tandem-transformations of **1** in alcohols/aqueous medium in the presence of bases and nucleophiles. The electrochemical behavior of the 6-alkoxy-2-amino-4-ferrocenylpyridine-3,5-dicarbonitriles and 6-alkoxy-2-amino-4-ferrocenyl-3-ferrocenylmethyl-3,4-dihydropyridine-3,5-dicarbonitriles was studied.

## 2. Results and Discussion

### 2.1. Synthesis of 6-Alkoxy-2-amino-4-ferrocenylpyridine-3,5-dicarbonitriles and 6-alkoxy-2-amino-4-ferrocenyl-3-ferrocenylmethyl-3,4-dihydropyridine-3,5-dicarbonitriles

**Scheme 1:**
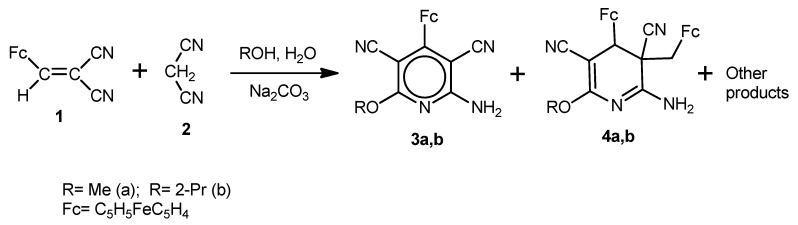
Reaction of 2-cyano-3-ferrocenylacrylonitrile (**1**) with malononitrile (**2**) in the presence of Na_2_CO_3_.

We found that two competitive processes occur upon reaction of 2-cyano-3-ferrocenylacrylonitrile (**1**) with malononitrile (**2**) in MeOH/H_2_O or 2-PrOH/H_2_O medium in the presence of Na_2_CO_3_, *viz*, formation of 6-alkoxy-2-amino-4-ferrocenylpyridine-3,5-dicarbonitriles **3a**,**b** (multi-component condensation) and of cyclodimeric products **4a**,**b** (multi-component cyclodimerization). In addition, other minor reaction products of the starting compounds were also isolated, however their structures could not be established from their ^1^H and ^13^C-NMR spectroscopy and mass spectrometry data (Scheme 1).

The compounds **3a**,**b** and **4a**,**b** were isolated by column chromatography on alumina and their structures were characterized by IR and NMR spectroscopy, mass spectrometry, and elemental analysis (see Experimental section). According to the ^1^H- and ^13^C-NMR data, the cyclodimerization of **1** occurs with high diastereoselectivity, and compounds **4a** and **4b** were isolated as a single diastereomeric form. One cannot rule out the formation of minor diastereomeric products; however they could not be isolated and characterized.

**Figure 1:**
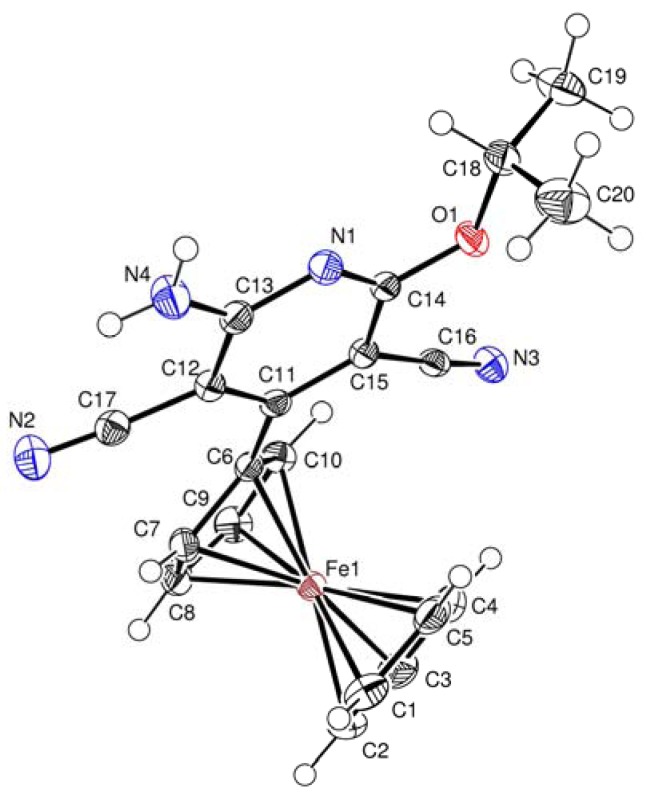
Crystal structure of **3b**.

**Figure 2:**
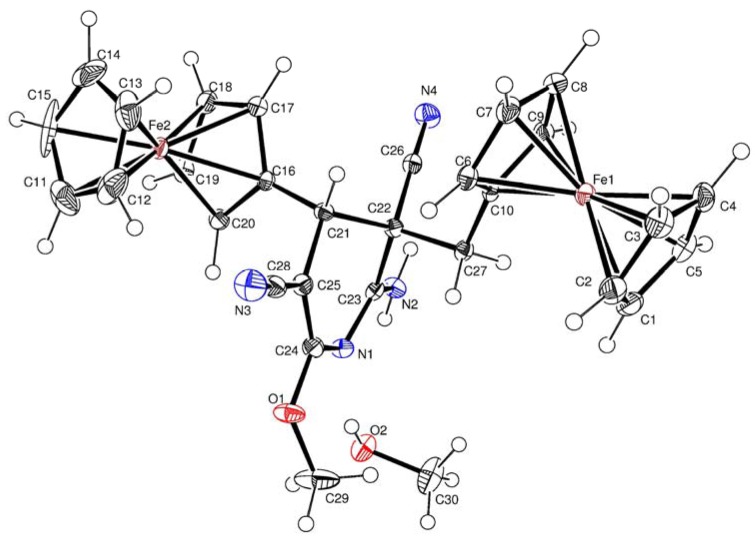
Crystal structure of **4a**.

**Figure 3:**
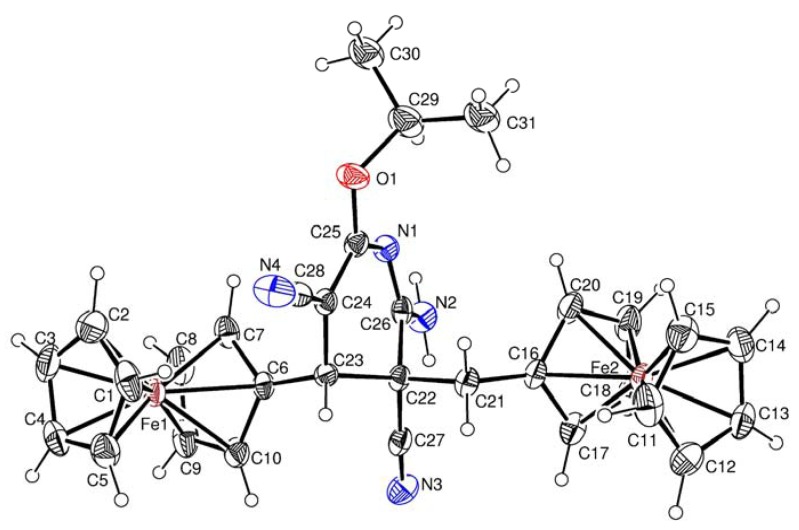
Crystal structure of **4b**.

The molecular structures of compounds **3b**, **4a** and **4b** were determined by X-ray diffraction analysis of their single crystals. The general views of molecules **3b**, **4a**, and **4b** are shown in Figure 1 Figure 2 Figure 3, respectively, while the principal geometric parameters are listed in Table 1.

X-Ray diffraction analysis confirmed the aromatic ferrocenylpyridine structure for compound **3b**, and diferrocenyl(dihydro)pyridine structures for compounds **4a** and **4b**. Central fragment of the molecule **3b** is a flat six-membered ring with one nitrogen atom. The N(1)-C(14) bond length in the compound **3b** is somewhat shorter [*d* = 1.318(2) Å] compared to the standard length (cf. *d* = 1.338 Å [24]). The bond lengths of the C–Fe and C–C bonds in the ferrocenyl substituents as well as the geometric parameters of the ferrocene sandwiches are close to standard values [25].

**Table 1:** Selected bond lengths and bond angles for compounds **3b**, **4a** and **4b**.

Selected bond lengths (Å)			Selected bond angles (°)			
3b						
N(1)-C(13)	1.346(2)		C(15)-C(11)-C(12)		116.21(15)	
N(1)-C(14)	1.318(2)		N(1)-C(13)-C(12)		122.50(16)	
N(4)-C(13)	1.337(2)		N(1)-C(14)-O(1)		120.55(15)	
N(3)-C(16)	1.1150(2)		N(1)-C(14)-C(15)		124.42(16)	
C(12)-C(13)	1.422(2)		C(11)-C(15)-C(14)		119.28(16)	
C(11)-C(12)	1.406(2)		C(13)-N(1)-C(14)		117.46(15)	
O(1)-C(14)	1.338(2)		N(4)-C(13)-N(1)		116.87(16)	
C(11)-C(15)	1.404(2)		C(14)-O(1)-C(18)		119.91(14)	
C(15)-C(14)	1.417(2)		C(17)-C(12)-C(13)		116.37(15)	
O(1)-C(18)	1.466(2)		C(12)-C(11)-C(6)		120.99(15)	
**4a**						
N(1)-C(23)	1.319(3)		N(1)-C(23)-C(22)		120.62(18)	
C(24)-N(1)	1.381(3)		N(1)-C(24)-C(25)		124.48(19)	
C(22)-C(23)	1.530(3)		N(1)-C(24)-O(1)		116.55(18)	
C(21)-C(22)	1.568(3)		C(23)-C(22)-C(21)		106.96(16)	
C(21)-C(25)	1.512(3)		C(22)-C(21)-C(25)		106.41(16)	
C(25)-C(24)	1.352(3)		C(21)-C(25)-C(24)		118.82(18)	
C(22)-C(27)	1.566(3)		N(1)-C(23)-N(2)		120.29(19)	
C(23)-N(2)	1.312(3)		N(2)-C(23)-C(22)		118.99(18)	
C(24)-O(1)	1.345(2)		C(27)-C (22)-C(26)		107.67(17)	
**4b**						
N(1)-C(25)	1.378(4)		C(24)-C(23)-C(22)		105.9(3)	
N(1)-C(26)	1.291(4)		N(4)-C(28)-C(24)		177.4(4)	
N(2)-C(26)	1.334(5)		N(2)-C(26)-C(22)		118.3(3)	
N(3)-C(27)	1.137(5)		N(3)-C(27)-C(22)		178.0(4)	
N(4)-C(28)	1.147(4)		C(26)-C(22)-C(23)		103.3(3)	
C(22)-C(21)	1.559(5)		C(24)-C(25)-N(1)		124.1(3)	
C(22)-C(26)	1.532(5)		N(2)-C(26)-N(1)		119.3(3)	
C(23)-C(22)	1.569(4)		C(21)-C(22)-C(23)		111.6(3)	
C(24)-C(25)	1.359(5)		C(26)-N(1)-C(25)		117.0(3)	
C(23)-H(23)	0.9800		C(22)-C(26)-N(1)		122.4(3)	
C(25)-O(1)	1.345(4)		O(1)-C(25)-N(1)		116.8(3)	
C(29)-O(1)	1.443(5)		C(27)-C(22)-C(23)		109.9(3)	
C(24)-C(23)	1.510(5)		C(26)-C(22)-C(23)		106.3(3)	

Key elements of the molecules **4a** and **4b** are the central six-membered ring with one nitrogen atom in the half-chair conformation. The N(1)-C(23) (for **4a**) and N(1)-C(26) (for **4b**) bond lengths are equal to *d* = 1.319(3) Å, and *d* = 1.291(4) Å, respectively. The ferrocenyl and ferrocenylmethyl substituents at C-4 and C-5 of **4a** and **4b** are *trans* oriented relative to the 6-membered cycle.

**Scheme 2:**
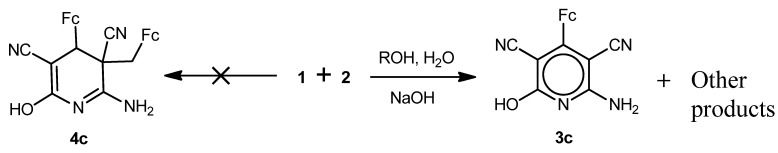
Reaction of 2-cyano-3-ferrocenylacrylonitrile (**1**) with malononitrile (**2**) in the presence of NaOH.

**Scheme 3:**
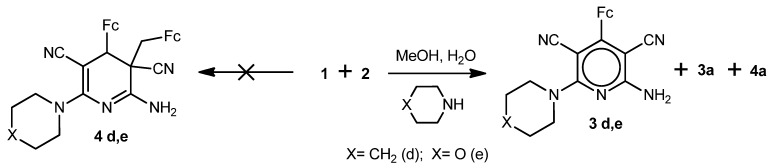
Reaction of 2-cyano-3-ferrocenyl-acrylonitrile (**1**) with malononitrile (**2**) in the presence of piperidine or morpholine.

We found further that 2-cyano-3-ferrocenylacrylonitrile (**1**) reacted analogously with malononitrile (**2**) in MeOH/H_2_O medium in the presence of NaOH, piperidine or morpholine to give compounds **3a**,**c**–**e** and **4a** (Scheme 2 and Scheme 3). Cyclodimeric products **4c**,**d**,**e** with hydroxy-, piperidino- or morpholino-substituents were not detected (Scheme 2 and Scheme 3). As in the case of reaction of **1** with **2** in the presence Na_2_CO_3_, the polymerization products of the starting compounds were also present in minor quantities.

Both reaction mixtures were separated by column chromatography on alumina, and the structures of the isolated products were characterized by IR, ^1^H and ^13^C-NMR spectroscopy, mass spectrometry, and elemental analysis. These physicochemical characterizations of compounds **3c**–**e** corroborate completely their structures.

**Scheme 4:**
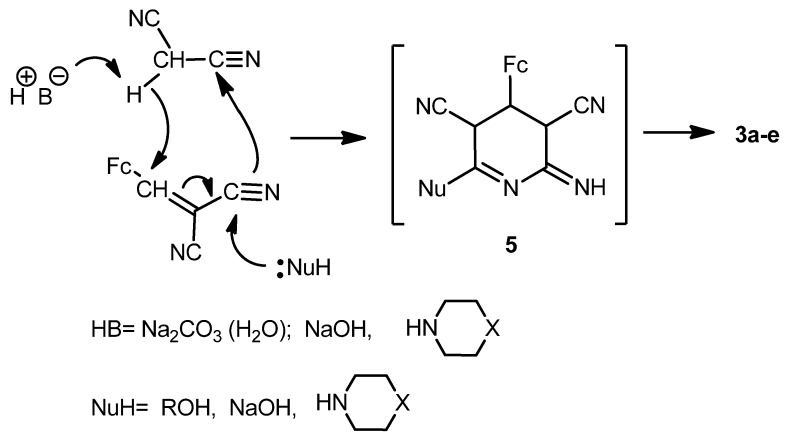
Possible mechanism for the formation of compounds **3a**–**e**.

The formation of 2-amino-4-ferrocenylpyridine-3,5-dicarbonitriles **3a**-**e** in the presence of bases and nucleophiles proceeds, in our opinion, via multi-component condensation reaction [26] (Scheme 4). Possibly the intermediate **5** is generated in one step and then transformed into pyridines **3a**–**e**.

**Scheme 5:**
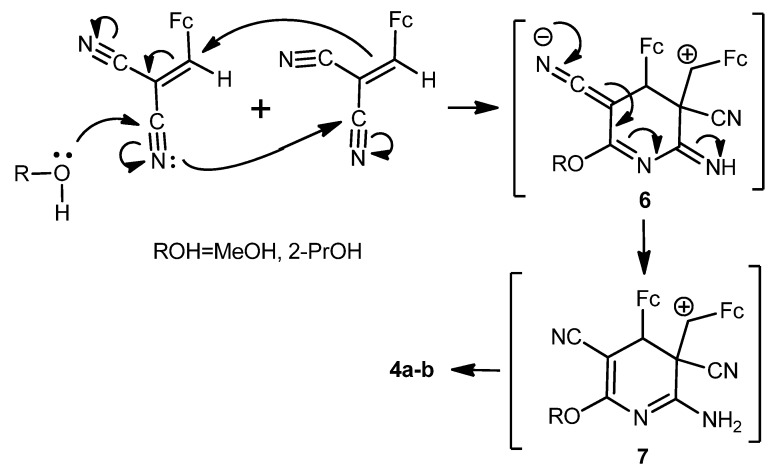
Possible mechanism for the formation of compounds **4a**,**b**.

A tentative mechanism for the formation of the diferrocenyl(dihydro)pyridine-3,5-dicarbonitriles **4a**,**b** is represented in Scheme 5.

To verify the mechanism described in Scheme 5 above, the cyclodimerization of 2-cyano-3-ferrocenylacrylonitrile (**1**) was carried out under identical conditions in 2-propanol in the presence of water and Na_2_CO_3_. The product of the cyclodimerization, 2-amino-4-ferrocenyl-3-ferrocenylmethyl-6-isopropoxy-3,4-dihydropyridine-3,5-dicarbonitrile (**4b**), was obtained with ~27% yield. Thus, cyclodimerization of compound **1** represents a novel type of the three-component anomalous reaction of [4+2]-cycloadition, absolutely different from the Diels-Alder reaction.

### 2.2. Electrochemistry

**Figure 4:**
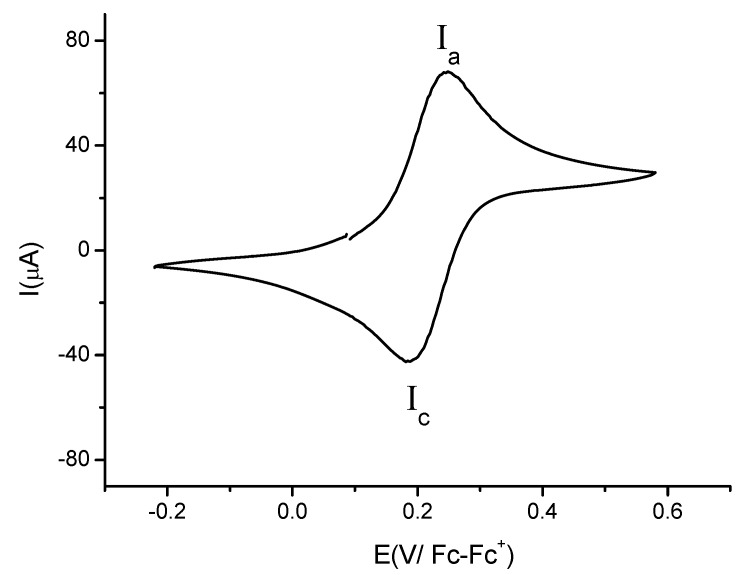
Cyclic voltammogram of compound **3b** in the presence of 0.1 M TBABF_4_ in MeCN. Scan rate 0.1 V·s^−1^. The working electrode used was platinum.

Figure 4 shows a typical voltammogram of compound **3b** recorded from open circuit potential to positive direction using a platinum electrode. It was observed one oxidation signal **I_a_** with anodic peak potential value *E*_pa_(**I_a_**) = 0.247 V/Fc-Fc^+^ and, one reduction signal **I_c_**, with cathodic peak potential value *E*_pc_(**I_c_**) = 0.184 V/Fc-Fc^+^. The Δ*E*p = 0.063 was independent of scan rate (from 0.1 to 1 V·s^−1^). The cathodic peak current and the anodic peak current were proportional to *v*^1/2^, indicating that **I** is a diffusion-controlled process [27]. The evidence presented above suggests that process **I** can be attributed to the reversible electron transfer for the ferrocene moiety Fc-Fc^+^. The formal potential electrode value was E^0'^ = 0.215 V/Fc-Fc^+^, estimated as E^0'^ = 1/2(E_pa_ + E_pc_). The electrochemical behaviour of compound **3d** is very similar to that observed for **3b**. There are slight changes in peak potential values: *E*_pa_(**I_a_**) = 0.241 V/Fc-Fc^+^, *E*_pc_(**I_c_**) = 0.174 V/Fc-Fc^+^, Δ*E*p = 0.067 V and E^0'^ = 0.207 V/Fc-Fc^+^.

**Figure 5:**
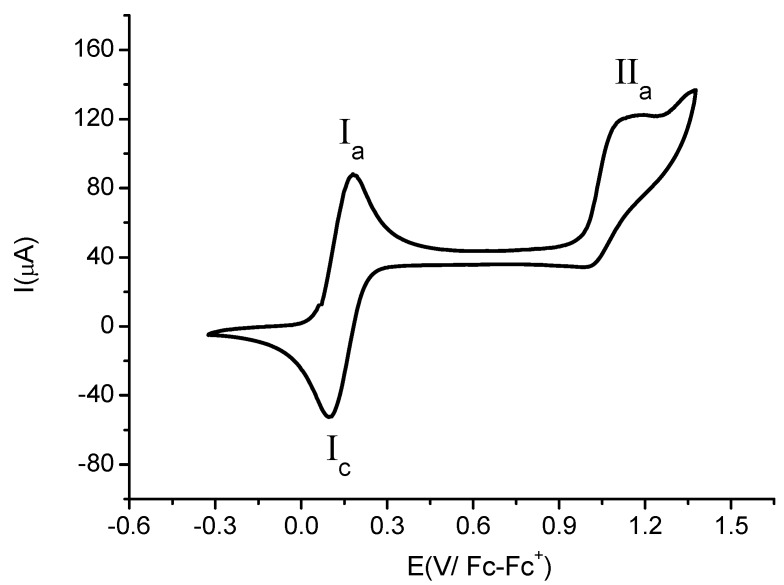
Cyclic voltammogram of compound **4b** in the presence of 0.1 M TBABF_4_ in MeCN. The scan rate 0.10 V·s^−1^. The working electrode used was platinum.

Figure 5 shows a cyclic voltammogram of compound **4b**. When the scan was started from open circuit potential to positive direction two oxidation signals (**I**_a_) and (**II**_a_) were observed. The anodic peak potentials values for these signals are *E*_pa_(**I**_a_) = 0.198 V/Fc-Fc^+^ and *E*_pa_(**II**_a_) = 1.149 V/Fc-Fc^+^. When the cycle was complete only one reduction signal **I**_c_ (related to the oxidation process **I**_a_) was observed. The estimated cathodic peak potential value was *E*_pc_(**I**_c_) = 0.099 V/Fc-Fc^+^. Despite the use of different scan rates (0.1 V·s^−1^–1.0 V·s^−1^) in the voltammetric experiments, the product of the electrochemical reduction in the process **IIa** was not detected. This result points out the absence of electronic communication between the two proximal ferrocenyl centres, which is contrary to the observations reported recently [28], where the communication between ferrocenyl fragments was detected in 3,5-diferrocenylpyridine. The electrochemical process **I** is attributed to the ferrocene moiety at the *para* position to the nitrogen atom of the heterocycle, Fc*_para_*^−^/Fc*_para_*^+^. The estimated formal potential electrode value was E^0'^ = 0.1485 V/Fc-Fc^+^.

The second oxidation process (**IIa**) is related to the ferrocene moiety at the *meta* position to the nitrogen atom of the heterocycle (Fc*_meta_*/Fc*_meta_*^+^) with high positive electronic density due to its proximity to the CN group. The absence of the reduction signal in the process **II** could be attributed to a low stabilization of the electro-generated dication (Fc^+^_para_-Fc*_meta_*^+^) by the solvent [29,30]. This fact was confirmed when the experiment was performed in a coordinative solvent such as DMSO, where electrochemical response becomes more irreversible.

## 3. Experimental

### 3.1. General

All the solvents were dried according to the standard procedures and were freshly distilled before use [31]. Column chromatography was carried out on alumina (Brockmann activity III). The ^1^H and ^13^C-NMR spectra were recorded on a Unity Inova Varian spectrometer (300 and 75 MHz, Palo Alto, CA, USA) for solutions in CDCl_3_, with Me_4_Si as the internal standard. The IR spectra were measured on a Spectrophotometer FT-IR (Spectrum RXI Perkin Elmer instruments, Waltham, MA, USA) using KBr pellets. The mass spectra were obtained on a Varian MAT CH-6 instrument (EI MS, 70 eV). Elementar Analysensysteme LECO CHNS-900 (St. Joseph, MI, USA) was used for elemental analysis.

The electrochemical behavior of compounds **3b**, **3d** and **4b** was explored with a Biologic SP-50 (Grenoble, France) potentiostat/galvanostat. The current interrupt method was used for *iR* compensation during all the experiments. The sample concentration employed was ca. 1 mM in acetonitrile in the presence of 0.1 M *tetra*-*N*-butylammonium tetrafluoroborate (TBABF_4_). A platinum disk and a platinum wire were used as working electrode and counter-electrode, respectively. A silver wire was used as a pseudo reference electrode. All solutions were bubbled with nitrogen 5 minutes prior each measurement. Cyclic voltammetry experiments were initiated from open circuit potential (E_ocp_) to positive direction, using scan rates from 0.1 to 1.0 V·s^−1^. All potentials were reported *versus* the couple Fc/Fc^+^ according to IUPAC convention [32]. The following reagents were purchased from Aldrich (Toluca, Mexico): ferrocenecarboxaldehyde, 99%; malononitrile, 99%; methyl alcohol, 99.9%; 2-propanol, 99.9%; morpholine, 99+%; piperidine, 99%. 2-Cyano-3-ferrocenylacrylonitrile (**1**) was prepared by condensation of ferrocenecarbaldehyde with malononitrile in benzene in the presence of piperidinium acetate [33]. The physical and ^1^H-NMR spectroscopic characteristics of compound **1** were in accordance with the literature data [34].

*Reactions of 2-cyano-3-ferrocenylacrylonitrile* (**1**) *with malononitrile* (**2**) *in the presence of Na_2_CO_3_*. A mixture of compound **1** (1.13 g, 5.0 mmol), malononitrile **2** (0.4 g, 6.0 mmol), methanol or 2-propanol (100 mL), H_2_O (10 mL) and Na_2_CO_3_ (0.5 g, 5.0 mmol) was stirred and refluxed for 8 h. The solvents were removed *in vacuo* and the residue was dissolved in dichloromethane (50 mL). The solution was mixed with Al_2_O_3_ (activity III, 20 g) and the solvent was evaporated in air. This sorbent was applied onto a column with Al_2_O_3_ (the height of alumina is *ca*. 20 cm) and the reaction products were eluted from the column first with petroleum ether, then with a 2:1 hexane–dichloromethane to give compounds **3a**,**b**, **4a**,**b** and polymeric compounds.

*2-Amino-4-ferrocenyl-6-methoxypyridine-3,5-dicarbonitrile* (**3a**). Red crystals, yield 0.93 g (52%), m.p. 162–163 °C; IR (KBr): 425, 497, 509, 575, 812, 843, 911, 1004, 1044, 1107, 1185, 1223, 1259, 1295, 1321, 1340, 1386, 1424, 1468, 1482, 1541, 1557, 1613, 2212, 2216, 2321, 2982, 3101, 3226, 3372, 3462 cm^−1^; ^1^H-NMR: 4.00 (s, 3H, CH_3_), 4.28 (s, 5H, C_5_H_5_), 4.59 (m, 2H, C_5_H_4_), 5.21 (m, 2H, C_5_H_4_), 5.57 (bs, 2H, NH_2_); ^13^C-NMR: 55.43 (CH_3_), 71.00 (C_5_H_5_), 70.49, 71.17 (C_5_H_4_), 84.91 (C*_ipso_*Fc), 116.27, 117.60 (2CN), 160.17, 160.43, 160.52, 164.88, 167.50 (5C); MS: *m/z* 358 [M]^+^. Anal. Calcd. for C_18_H_14_FeN_4_O: C 60.36, H 3.94, Fe 15.60, N 15.63. Found: C 60.45, H 4.04, Fe 15.46, N 15.49.

*2-Amino-4-ferrocenyl-6-isopropoxypyridine-3,5-dicarbonitrile* (**3b**). Red crystals, yield 0.9 g (48%), m.p. 176–177 °C; IR (KBr): 425, 502, 541, 584, 813, 845, 912, 1003, 1044, 1106, 1185, 1253, 1296, 1322, 1334, 1365, 1383, 1425, 1477, 1483, 1542, 1556, 1612, 2200, 2217, 2325, 2979, 3103, 3224, 3369, 3459 cm^−1^; ^1^H-NMR: 1.39 (d, 6H, 2CH_3_, *J* = 6.3 Hz), 4.28 (s, 5H, C_5_H_5_), 4.57 (m, 2H, C_5_H_4_), 5.20 (m, 2H, C_5_H_4_), 5.32 (m, 1H, CH, *J* = 6.3 Hz), 5.53 (bs, 2H, NH_2_); ^13^C-NMR: 21.95 (2CH_3_), 71.16 (CH), 71.01 (C_5_H_5_), 70.51, 71.07 (C_5_H_4_), 82.05 (C*_ipso_*Fc), 116.32, 117.77 (2CN), 160.14, 160.21, 161.56, 165.38, 166.86 (5C); MS: *m/z* 386 [M]^+^. Anal. Calcd. for C_20_H_18_FeN_4_O: C 62.20, H 4.70, Fe 14.46, N 14.50. Found: C 62.31, H 4.63, Fe 14.58, N 14.67.

*2-Amino-4-ferrocenyl-3-ferrocenylmethyl-6-methoxy-3,4-dihydropyridine-3,5-dicarbonitrile* (**4a**). Yellow crystals, yield 0.25 g (18%), m.p. dec. *ca*. 272 °C; IR (KBr): 484, 559, 691, 799, 811, 1002, 1029, 1041, 1103, 1190, 1235, 1282, 1321, 1371, 1387, 1452, 1472, 1534, 1550, 1597, 1641, 1663, 2217, 2225, 3090, 3321, 3429 cm^−1^; ^1^H-NMR: 2.91 (d, 1H, CH_2_, *J* = 14.1 Hz), 3.10 (d, 1H, CH_2_, *J* = 14.1 Hz), 3.47 (s, 1H, CH), 3.91 (s, 3H, CH_3_), 4.18 (s, 5H, C_5_H_5_), 4.27 (s, 5H, C_5_H_5_), 3.87 (m, 1H, C_5_H_4_), 4.08 (m, 1H, C_5_H_4_), 4.12 (m, 1H, C_5_H_4_), 4.17 (m, 1H, C_5_H_4_), 4.22 (m, 2H, C_5_H_4_), 4.23 (m, 1H, C_5_H_4_), 4.41 (m, 1H, C_5_H_4_), 5.59 (bs, 2H, NH_2_); ^13^C-NMR: 42.19 (CH_2_), 55.13 (CH_3_), 63.61 (CH), 69.23, 69.79 (2C_5_H_5_), 67.84, 68.36, 68.75, 68.99, 69.12, 69.56, 69.97, 70.44 (2C_5_H_4_), 80.21, 82.45 (2C*_ipso_*Fc), 119.21, 120.50 (2CN), 64.45, 160.62, 164.86, 165.72 (4C); MS: *m/z* 558 [M]^+^. Anal. Calcd. for C_29_H_26_Fe_2_N_4_O: C 62.40, H 4.70, Fe 20.01, N 10.03. Found: C 62.29, H 4.61, Fe 19.89, N 10.12.

*2-Amino-4-ferrocenyl-3-ferrocenylmethyl-6-isopropoxy-3,4-dihydropyridine-3,5-dicarbonitrile* (**4b**). Yellow crystals, yield 0.29 g (19%), m.p. dec. *ca*. 302 °C; IR (KBr): 483, 553, 682, 721, 783, 821, 915, 1001, 1026, 1042, 1106, 1142, 1181, 1249, 1294, 1316, 1355, 1371, 1383, 1423, 1475, 1544, 1585, 1629, 2191, 2300, 2930, 2978, 3095, 3241, 3335, 3466 cm^−1^; ^1^H-NMR: 1.39 (d, 6H, 2CH_3_, *J* = 6.0 Hz), 2.90 (d, 1H, CH_2_, *J* = 13.8 Hz), 3.09 (d, 1H, CH_2_, *J* = 13.8 Hz), 3.81 (s, 1H, CH), 4.13 (s, 5H, C_5_H_5_), 4.28 (s, 5H, C_5_H_5_), 4.08 (m, 1H, C_5_H_4_), 4.16 (m, 2H, C_5_H_4_), 4.23 (m, 2H, C_5_H_4_), 4.28 (m, 2H, C_5_H_4_), 4.42 (m, 1H, C_5_H_4_), 5.07 (m, 1H, CH, *J* = 6.0 Hz), 5.54 (bs, 2H, NH_2_). ^13^C-NMR: 21.94 (2CH_3_), 36.96 (CH_2_), 41.40, 66.96 (2CH), 69.04, 69.56 (2C_5_H_5_), 68.20, 68.54, 68.90, 68.94, 69.09, 69.84, 70.38, 71.52 (2C_5_H_4_), 79.25, 83.68 (2C*_ipso_*Fc), 119.16, 120.49 (2CN), 51.28, 160.43, 165.37, 166.82 (4C); MS: *m/z* 586 [M]^+^. Anal. Calcd. for C_31_H_30_Fe_2_N_4_O: C 63.51, H 5.16, Fe 19.05, N 9.55. Found C 63.67, H 5.03, Fe 19.13, N 9.41.

*Reaction of 2-cyano-3-ferrocenylacrylonitrile* (**1**) *with malononitrile* (**2**) *in the presence of NaOH*. The reaction of compound **1** (1.13 g, 5.0 mmol) with malononitrile **2** (0.4 g, 6.0 mmol) and 0.4 g NaOH in methanol (100 mL) and H_2_O (10 mL) was carried out under conditions described above; subsequent chromatography afforded **3a** (15%), **3c** and **4a** (12%). 

*2-Amino-4-ferrocenyl-6-hydroxypyridine-3,5-dicarbonitrile* (**3c**). Red crystals, yield 0.98 g (57%), m.p. 146–147 °C; IR (KBr): 423, 495, 508, 580, 814, 839, 910, 1004, 1042, 1103, 1181, 1242, 1290, 1321, 1340, 1378, 1420, 1468, 1481, 1540, 1554, 1612 2211, 2221, 2327, 2985, 3109, 3219, 3371, 3489, 3670 cm^−1^; ^1^H-NMR: 4.27 (s, 5H, C_5_H_5_), 4.58 (m, 2H, C_5_H_4_), 5.21 (m, 2H, C_5_H_4_), 5.60 (bs, 2H, NH_2_), 5.73 (bs, 1H, OH); ^13^C-NMR: 71.05 (C_5_H_5_), 70.54, 71.22 (C_5_H_4_), 82.50 (C*_ipso_*Fc), 116.31, 117.61 (2CN), 157.70, 160.26, 160.82, 161.52, 167.58 (5C); MS: *m/z* 344 [M]^+^. Anal. Calcd. for C_17_H_12_FeN_4_O: C 59.33, H 3.52, Fe 16.23, N 16.27. Found: C 59.24, H 3.47, Fe 16.09, N 16.18.

*Reactions of 2-cyano-3-ferrocenylacrylonitrile* (**1**) *with malononitrile* (**2**) *in the presence of amines*. A solution of compounds **1** (5.0 mmol) and **2** (6.0 mmol), piperidine or morpholine (2.0 mL) in methanol (100 mL) was stirred for 6 h at 60 °C. The reaction mixture was evaporated *in vacuo*, and the residue was subjected to TLC on SiO_2_ (hexane–dichloromethane, 2:1) to give compounds **3a** (~20%, *Rf* = 0.78), **4a** (~9%, *Rf* = 0.67) and **3d**,**e** (58–61%, *Rf* = 0.35–0.54).

*2-Amino-4-ferrocenyl-6-piperidinopyridine-3,5-dicarbonitrile* (**3d**). Red crystals, yield 1.25 g (61%), m.p. 182–183 °C; IR (KBr): 416, 481, 503, 585, 814, 912, 1001, 1019, 1100, 1177, 1251, 1289, 1311, 1343, 1392, 1425, 1464, 1472, 1546, 1553, 1612, 2217, 2226, 2334, 2973, 3101, 3239, 3388, 3469 cm^−1^; ^1^H-NMR: 1.70 (m, 2H, CH_2_), 1.83 (m, 4H, 2 CH_2_), 3.18 (m, 4H, 2CH_2_), 4.32 (s, 5H, C_5_H_5_), 4.56 (m, 2H, C_5_H_4_), 5.05 (m, 2H, C_5_H_4_), 5.34 (bs, 2H, NH_2_); ^13^C-NMR: 24.55 (CH_2_), 26.05 (2CH_2_), 49.83 (2CH_2_), 70.86 (C_5_H_5_), 70.66, 70.90 (C_5_H_4_), 83.00 (C*_ipso_*Fc), 118.52, 119.29 (2CN), 160.51 (2C), 155.51, 160.74, 163.32 (3C); MS: *m/z* 411 [M]^+^. Anal. Calcd. for C_22_H_21_FeN_5_: C 64.25, H 5.15, Fe 13.58, N 17.02. Found: C 64.33, H 5.07, Fe 13.61, N 16.89.

*2-Amino-4-ferrocenyl-6-morpholinopyridine-3,5-dicarbonitrile* (**3e**). Red crystals, yield 1.20 g (58%), m.p. 190–192 °C; IR (KBr): 432, 491, 512, 591, 815, 861, 908, 1002, 1041, 1101, 1120, 1215, 1251, 1299, 1312, 1340, 1396, 1442, 1470, 1510, 1567, 1621, 1692, 2212, 2227, 2989, 3138, 3278, 3363, 3476 cm^−1^; ^1^H-NMR: 3.26 (m, 4H, 2CH_2_), 3.80 (m, 4H, 2CH_2_), 4.37 (s, 5H, C_5_H_5_), 4.68 (m, 2H, C_5_H_4_), 5.01 (m, 2H, C_5_H_4_), 5.65 (bs, 2H, NH_2_); ^13^C-NMR: 47.09 (2CH_2_), 50.98 (2CH_2_), 70.99 (C_5_H_5_), 67.17, 70.66 (C_5_H_4_), 80.35 (C*_ipso_*Fc), 116.03, 118.31 (2CN), 157.68 (2C), 155.25, 161.21. 168.65 (3C); MS: *m/z* 413 [M]^+^. Anal. Calcd. for C_21_H_19_FeN_5_O: C 61.04, H 4.63, Fe 13.52, N 16.94. Found: C 60.94, H 4.48, Fe 13.44, N 17.08.

*Chemical transformations of 2-cyano-3-ferrocenylacrylonitrile* (**1**) *in the presence of 2-PrOH, H_2_O and Na_2_CO_3_*. A mixture of compound **1** (1.13 g, 5.0 mmol), 2-propanol (60 mL), H_2_O (10 mL) and Na_2_CO_3_ (1.0 g, 10mmol) was stirred for 12 h at 80 °C. The reaction mixture was worked up as described above, subsequent chromatography on Al_2_O_3_ gave compounds **3b** (25%) and **4b** (27%), respectively, and polimeric compounds.

### 3.2. Crystal Structures of ***3b***, ***4a*** and ***4b***

Single crystals of **3b** and **4b** were obtained by crystallization from chloroform, while crystals of **4a** were obtained by crystallization from methanol. The unit cell parameters and the X-ray diffraction intensities were recorded on a Gemini (detector Atlas CCD, Cryojet N_2_, Loveland, CO, USA) diffractometer. The structures of compounds **3b**, **4a** and **4b** were solved by the direct method (SHELXS-97 [35]) and refined using full-matrix least-squares on F^2^.

*Crystal data for C_20_H_18_FeN_4_O* (**3b**): M = 386.23 g·mol^−1^, orthorhombic P bca, *a* = 12.4557(8), *b* = 14.9714(6), *c* = 18.4217(7) Å, α = 90, β = 90, γ = 90°, V = 3435.3(3) Å^3^, T = 130(2) K, Z = 8, ρ = 1.494 Mg/m^3^, wavelength 1.71073 Å, F(000) = 1,600, absorption coefficient 0.895 mm^−1^, index ranges −15 ≤ h ≤ 15, −18 ≤ k ≤ 17, −23 ≤ l ≤ 23, scan range 3.54 ≤ *θ* ≤ 26.73°, 3633 independent reflections, R_int_ = 0.0326, 26385 total reflections, 243 refinable parameters, final R indices [I > 2σ(I)] R_1_ = 0.0301, wR_2_ = 0.0705, R indices (all data) R_1_ = 0.0398, wR_2_ = 0.0775, goodness-of-fit on F^2^ 1.074, largest difference peak and hole 0.555/−0.310 eÅ^−3^.

*Crystal data for C_29_H_26_Fe_2_N_4_O·CH_3_OH* (**4a**): M = 590.28 g·mol^−1^, monoclinic P21/n, *a* = 13.1816(4), *b* = 10.0587(2), *c* = 20.4566(6) Å, α = 90, β = 107.323(3), γ = 90°, V = 2589.31(12) Å^3^, T = 130(2) K, Z = 4, ρ = 1.514 Mg/m^3^, wavelength 1.71073 Å, F(000) = 1,224, absorption coefficient 1.157 mm^−1^, index ranges −13 ≤ h ≤ 16, −12 ≤ k ≤ 12, −25 ≤ l ≤ 25, scan range 3.62 ≤ *θ* ≤ 26.05°, 5113 independent reflections, R_int_ = 0.0387, 18671 total reflections, 354 refinable parameters, final R indices [I > 2σ(I)] R_1_ = 0.0323, wR_2_ = 0.0720, R indices (all data) R_1_ = 0.0441, wR_2_ = 0.0781, goodness-of-fit on F^2^ 1.034, largest difference peak and hole 0.419/−0.337 eÅ^−3^.

*Crystal data for C_31_H_30_Fe_2_N_4_O* (**4b**): M = 586.29 g·mol^−1^, triclinic P-1, *a* = 10.5168(8), *b* = 11.7533(9), *c* = 12.4547(10) Å, α = 90.551(6), β = 111.455(7), γ = 107.402(7)°, V = 1354.90(18) Å^3^, T = 293(2) K, Z = 2, ρ = 1.437 Mg/m^3^, wavelength 1.71073 Å, F(000) = 608, absorption coefficient 1.102 mm^−1^, index ranges −12 ≤ h ≤ 11, −14 ≤ k ≤ 14, −11 ≤ l ≤ 15, scan range 3.55 ≤ *θ* ≤ 26.06°, 5346 independent reflections, R_int_ = 0.0500, 9894 total reflections, 344 refinable parameters, final R indices [I > 2σ(I)] R_1_ = 0.0558, wR_2_ = 0.1336, R indices (all data) R_1_ = 0.0785, wR_2_ = 0.1551, goodness-of-fit on F^2^ 1.050, largest difference peak and hole 0.877/−0.748 eÅ^−3^. CCDC-878738 (for **3b**), CCDC–878739 (for **4a**) and CCDC-878741 (for **4b**) contain the supplementary crystallographic data for this paper. These data can be obtained free of charge at www.ccdc.cam.ac.uk/const/retrieving.html [or from the Cambridge Crystallographic Data Centre, 12, Union Road, Cambridge DB2 1EZ, UK; Fax: (internat.) +44-1223-336-033; Email: deposit@ccdc.cam.ac.uk]. 

## 4. Conclusions

The reaction of 2-cyano-3-ferrocenylacrylonitrile (**1**) with malononitrile (**2**) in a MeOH/H_2_O or 2-PrOH/H_2_O medium in the presence of Na_2_CO_3_, NaOH, piperidine or morpholine affords products of multi-component condensation: 6-alkoxy-2-amino-, 2-amino-6-hydroxy-, 2,6-diamino-4-ferrocenylpiridine-3,5-dicarbonitriles **3a**–**e**, respectively, as well as products of multi-component cyclodimerization: 6-alkoxy-2-amino-4-ferrocenyl-3-ferrocenylmethyl-3,4-dihydropyridine-3,5-dicarbonitriles **4a**,**b**. This method can be widely used in the synthesis of various pyridine derivatives with ferrocenyl substituents. The reactions described in this study should be of interest to synthetic, theoretical and practical organic chemists seeking ways to prepare functionalized ferrocenylpyridines. The electrochemical behavior of compounds **3b**, **3d** and **4b** was investigated by means of cyclic voltammetry. For **3b** and **3d** two electrochemical processes (**Ia**,**Ic**), attributed to the oxidation and reduction of the ferrocene moieties were found. On the other hand, for compound **4b** a double electron transfer for both ferrocene groups (**Ia**,**IIa**) and the electrochemical monogeneration of the dication species (**Ic**) were detected. 
